# Magnetic Resonance Spectroscopy May Help Diagnose Sporadic Meningioangiomatosis Associated With Meningioma: A Case Report

**DOI:** 10.3389/fneur.2022.912728

**Published:** 2022-07-11

**Authors:** Linfeng Liu, Feng Liang

**Affiliations:** Department of Neurosurgery, First Affiliated Hospital of Sun Yat-sen University, Guangzhou, China

**Keywords:** meningioangiomatosis, meningioma, magnetic resonance spectroscopy, meningioangiomatosis associated with meningioma, case report

## Abstract

Herein, we have presented the clinical features of meningioangiomatosis associated with meningioma, which is considered to be a rare neoplastic lesion. Magnetic resonance spectroscopy (MRS) demonstrated a remarkably decreased N-acetylaspartate peak and an increase in the choline peak of the lesion, suggesting neuronal injury and active cell proliferation. These findings substantially differed from those observed in the case of pure meningioangiomatosis.

## Introduction

Meningioangiomatosis (MA) is regarded as a benign hyperplastic tumor-like lesion characterized by meningovascular proliferation, with seizures being the most common clinical symptom. It occurs sporadically in children or young adults, accounting for ~80% of all cases (1). However, MA may occasionally occur in combination with meningioma (MA-M), and this component is associated with increased Ki-67 compared to MA, suggesting resemblance with a neoplasm with greater proliferative behavior and, consequently, more severe and intolerable symptoms (2). Thus, more aggressive resection is recommended for MA-M (3). However, neither pure MA nor MA-M have specific neuroradiological features or histopathological characteristics to aid differential diagnosis, making distinction difficult and clinical diagnosis complicated (4). Therefore, specific imaging examinations are necessary to enable the identification of this rare disease, and to ensure timely diagnosis and early intervention.

Magnetic resonance spectroscopy (MRS) was found to be particularly useful in this case because it demonstrated a remarkably decreased N-acetylaspartate (NAA) peak and increased choline (Cho) peak of the lesion, suggesting neuronal injury and active cell proliferation. However, to the best of our knowledge, only two cases of MRS for pure MA have been reported to date (5, 6). To aid early detection of this disorder, we present a case of sporadic MA-M in an 8-year-old boy and discuss the advantages and importance of MRS in the diagnosis of MA-M. This study was approved by the First Affiliated Hospital of Sun Yat-sen University, and consent for publication of this case report was obtained from the patient's parents.

## Case Presentation

An 8-year-old boy experienced paroxysmal dizziness for 5 years. Six months prior to admission to our hospital, he experienced intermittent tremors in the right upper extremity without any obvious predisposing factors. Gradually he developed generalized tonic-clonic seizures accompanied by right arm stiffness, generalized convulsion, aphasia, and tilting of the head and eyes.

**Figure 1:**
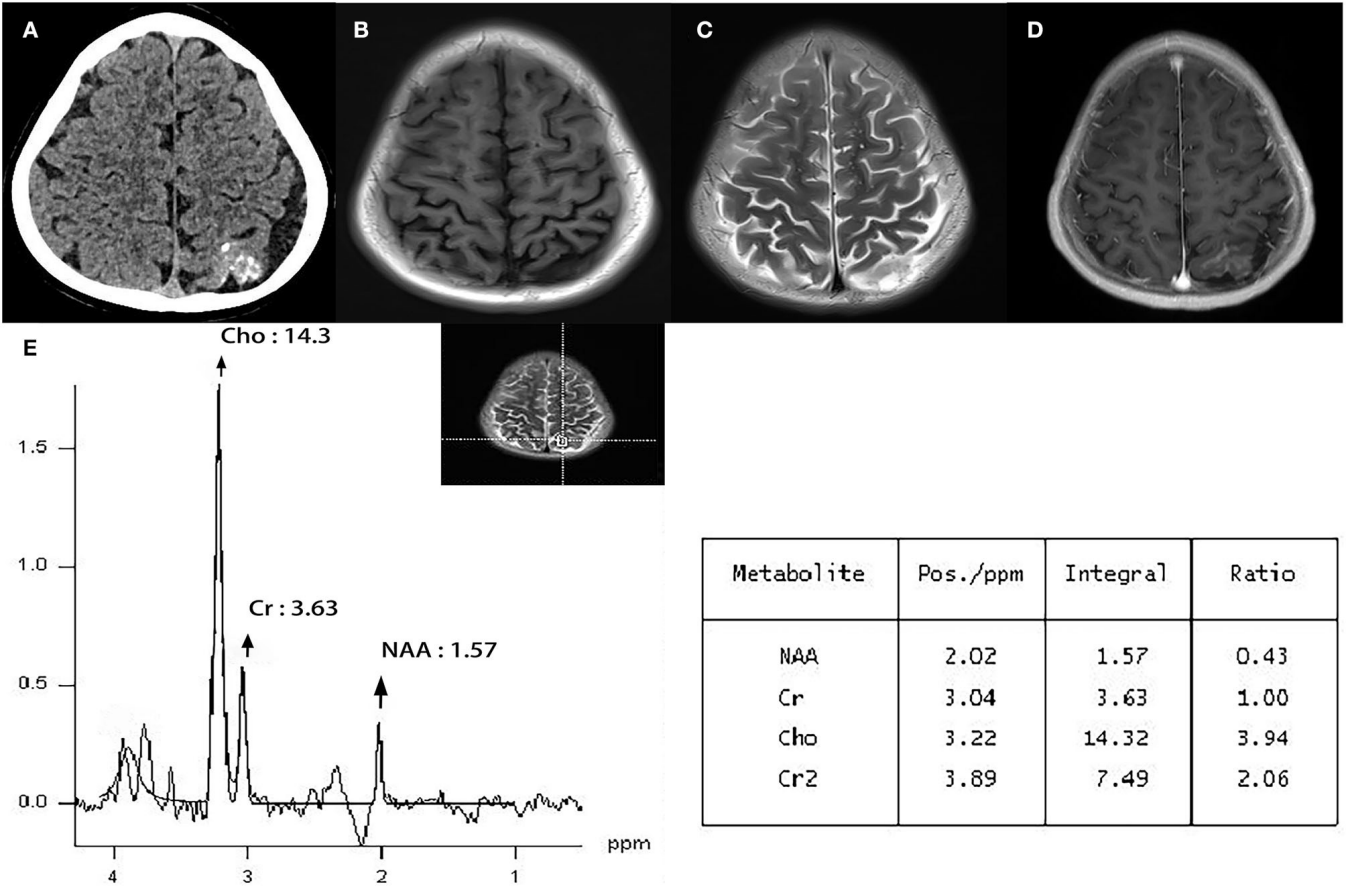
Plain CT demonstrated a hyperdense lesion with multiple calcifications and proliferating adjacent blood vessels in the left parietal lobe. **(A)** T1-weighted MRI showed hypointense irregular abnormal cortical and subcortical regions. **(B)** T2-weighted MRI demonstrated a hyperintense mass. **(C)** MR post gadolinium enhancement showed enhanced pia mater and cortex. **(D)** Magnetic resonance spectroscopy (MRS) revealed decreased NAA and increased Cho in the lesion. The Cho/Cr ratio was markedly elevated (3.94), while the NAA/Cr ratio was reduced (0.43) in the lesion area **(E)**.

He was admitted for diagnosis and treatment; no obvious abnormalities were observed upon physical and neurological examination. No personal or family history of neurological diseases was reported, but plain computed tomography (CT) demonstrated a hyperdense lesion with multiple calcifications and proliferating adjacent blood vessels in the left parietal lobe (Figure 1A). T1-weighted magnetic resonance imaging (MRI) showed a hypointense irregular abnormality in the cortical and subcortical regions (Figure 1B), whereas T2-weighted MRI demonstrated a hyperintense mass (Figure 1C). MR post gadolinium enhancement showed enhanced pia mater and cortex (Figure 1D). Furthermore, MRS revealed decreased NAA and increased Cho in the lesion (Figure 1E). When compared with the control area where the Cho/Cr and NAA/Cr ratios were 1.03 and 1.69, respectively, the Cho/Cr ratio was found to be markedly elevated (3.94), and the NAA/Cr was reduced (0.43) in the lesion area. The calculated Cho/NAA ratio was 19.12.

**Figure 2:**
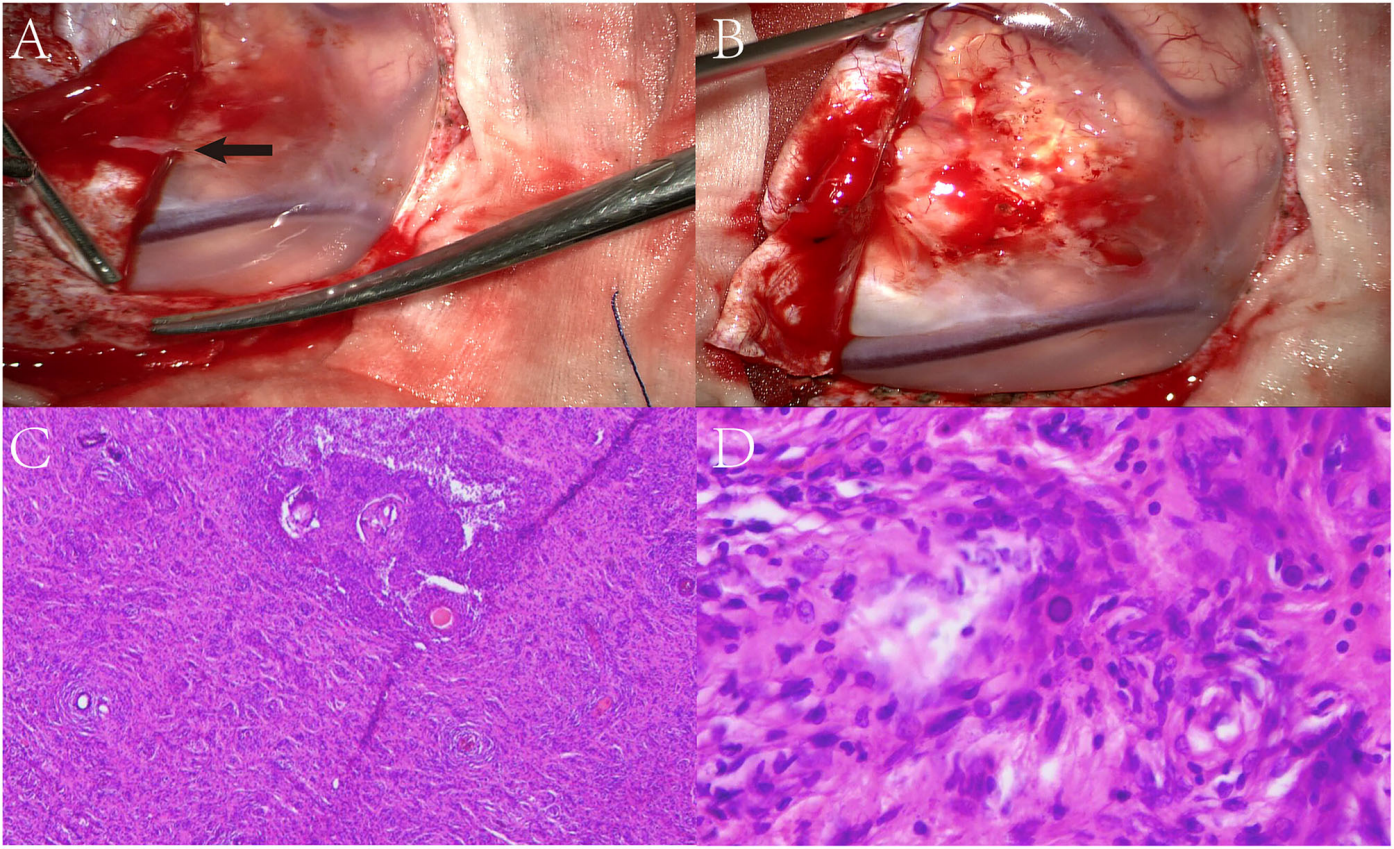
Surgical microscopic examination showed an ill-defined mass with a grayish-yellow surface. The overlying dura was involved (black arrow) and the surrounding tissue was slightly edematous **(A,B)**. Biopsy showed a diffused proliferation of spindle and oval cells with some psammoma bodies present in the cortex **(C,D)**.

The patient underwent a left parieto-occipital craniotomy, which revealed an ill-defined mass with a grayish-yellow surface. The overlying dura was determined to be involved, and the surrounding tissues were slightly edematous (Figures 2A,B). Intra-operative electrocorticography indicated that the epileptiform discharge was highly correlated with the lesion, which was completely removed along with the overlying dura. Electrocorticography was performed again to ensure no epileptiform discharges.

Microscopically, the lesion area was poorly circumscribed regarding normal brain tissue, and the presence of diffused proliferation of spindle and oval cells in addition to psammoma bodies was observed in the cortex. Extensive spindle-shaped cells were found to be arranged in a whorled pattern surrounding the vessels, and syncytial cells were present in local regions (Figures 2C,D). No obvious atypia or mitosis was observed.

Immunohistochemistry revealed epithelial membrane antigen (EMA) positive findings, and the Ki-67 index in the meningioma was 3%. NeuN showed focal neuronal arrangement disorder. Based on these results, the final diagnosis was MA-associated with WHO grade 1 fibrous meningioma. The patient was discharged without any adjuvant radiotherapy or chemotherapy and was seizure-free at the 3-month follow-up. Moreover, the elevated Cho peak decreased over the 3-month follow-up period, and the Cho/Cr and NAA/Cr ratios were 1.08 and 0.00, respectively.

## Discussion

The MA is commonly defined as a benign hyperplastic tumor-like lesion in the cerebral cortex, marked by meningeal and vascular proliferation. It has been found to be occasionally associated with MA-M, which is considered to be a neoplastic lesion (2), highlighting the need for more efficient and aggressive treatment measures. However, limited understanding of characteristic clinical manifestations complicates the distinction between MA and MA-M, making clinical diagnosis complex. A review of related literature revealed that MA is often associated with a hypointense region on T1-weighted MRI and a hyperintense area on T2-weighted images, whereas contrast-enhanced MRIs usually demonstrate irregular or homogeneous enhancement (7). This case report is consistent with these findings, suggesting that the possibility of MA should be considered when differentiating between calcified lesions or meningeal invasion.

At present, the most obvious differences between MA and MA-M reported in the literature include the incidence of seizures and symptom duration (2), with the latter being associated with a lower incidence of epilepsy and shorter duration. MA is considered to be a developmental non-neoplastic lesion with more well-differentiated cells, resulting in greater production of neurotransmitters that cause seizures. Conversely, MA-M represents a different kind of neoplastic disease, composed of poorly differentiated cells that are unable to effectively release neurotransmitters. However, these differences are insufficient to aid the clinical distinction between MA and MA-M, given the lack of explicit metrics.

The MRS is a non-invasive imaging technique that can help clinicians identify potentially malignant lesions (8). The Cho/Cr and Cho/NAA ratios of MRS exhibit high sensitivity about distinguishing benign and malignant intracranial lesions (9, 10). Lin et al. also proposed that the Cho/NAA ratios of MRS may serve as simple and practical measures for the confirmation of the grade of intracranial meningioma, before clinical decision-making (11). However, there is limited evidence on the use of MRS for the diagnosis of MA or MA-M.

In this case study, MRS indicated decreased NAA and increased Cho in the lesion, suggesting associated neuronal injury and active cell proliferation. The Cho/Cr and NAA/Cr ratios in the lesion area were 3.94 and 0.43, respectively, and this was in contrast to previous studies on MA that reported only a slight change in MRS. Nomura (5) reported a patient with pure MA who exhibited a decreased NAA peak and slightly increased Cho peak, with a spectrum pattern similar to that of low-grade astrocytoma (5). However, no malignant features were observed upon pathological examination. Another case report of a patient with pure MA revealed normal MRS results, indicating no specific neuronal loss (6) and suggesting that pure MA is a benign non-neoplastic lesion, in accordance with existing evidence (1, 12). Unfortunately, neither of these studies reported specific NAA/Cr, Cho/Cr, or Cho/NAA ratios. These results may reveal one of the key differences between the two diseases. MA-M is associated with neoplastic characteristics and often results in more severe symptoms (2, 12), suggesting that it is more likely to mimic the process of malignant invasion and contribute to an increase in the Cho/NAA ratio (13).

According to the previous literature, the spectrum of meningioma can show a peak at 1.47 or 1.50 ppm, which can be assigned to alanine (Ala) *in vitro* spectrum and *in vivo* spectrum, respectively (14). Ala is regarded as a metabolite characteristic of meningioma and is maintained through neoplastic transformation (15, 16). However, our spectrum results did not find an obvious Ala peak. That may be because the meningioma component in the lesion transforms from the MA component, resulting in different metabolic characteristics and spectrum of typical meningioma (2). It is a pity that *ex vivo* high-resolution magic angle spinning (HR-MAS) was not performed then, which can help better interpretation of the *in vivo* spectrum.

Moreover, the Cho/NAA ratio in the current case study was 19.12, indicating an invasive malignant lesion (11). Histopathological examination and immunohistochemistry demonstrated that the meningioma was benign, consistent with results of previous studies (3, 17), and we hypothesize that the invasive behavior of the MA component may have contributed to these findings. Because the vessels of MA could be embedded in a collagenous stroma and the MA component displayed an infiltrative pattern of growth-entrapping cortical elements, such as neurons (3). However, these explanations, being less convincing, can be attributed to the overlap between the spectrum of patterns of different diseases (18); the optimal cutoff points for the Cho/Cr and Cho/NAA ratios between MA and MA-M remain unclear due to insufficient evidence on MRS examination of MA or MA-M.

Therefore, although MRS may help distinguish MA-M before postoperative pathological diagnosis, more clinical data is essential to assess its value in this disease.

## Data Availability Statement

The original contributions presented in the study are included in the article/supplementary material, further inquiries can be directed to the corresponding author.

## Ethics Statement

The studies involving human participants were reviewed and approved by First Affiliated Hospital of Sun Yat-sen University. Written informed consent to participate in this study was provided by the participants' legal guardian/next of kin. Written informed consent was obtained from the minor(s)' legal guardian/next of kin for the publication of any potentially identifiable images or data included in this article.

## Author Contributions

LL: collecting the data and drafting the manuscript. FL: critical revisions of the manuscript. All authors participated in the article and approved the final version.

## Conflict of Interest

The authors declare that the research was conducted in the absence of any commercial or financial relationships that could be construed as a potential conflict of interest.

## Publisher's Note

All claims expressed in this article are solely those of the authors and do not necessarily represent those of their affiliated organizations, or those of the publisher, the editors and the reviewers. Any product that may be evaluated in this article, or claim that may be made by its manufacturer, is not guaranteed or endorsed by the publisher.
